# Renewable Chemicals: Dehydroxylation of Glycerol and Polyols

**DOI:** 10.1002/cssc.201100162

**Published:** 2011-08-22

**Authors:** Jeroen ten Dam, Ulf Hanefeld

**Affiliations:** [a] Gebouw voor Scheikunde, Afdeling Biotechnologie, Technische Universiteit DelftJulianalaan 136, 2628 BL Delft (The Netherlands), Fax: (+31) 15 278 1415 E-mail: u.hanefeld@tudelft.nl

**Keywords:** dehydroxylation, glycerol, hydrogenolysis, polyols, renewable chemicals

## Abstract

The production of renewable chemicals is gaining attention over the past few years. The natural resources from which they can be derived in a sustainable way are most abundant in sugars, cellulose and hemicellulose. These highly functionalized molecules need to be de-functionalized in order to be feedstocks for the chemical industry. A fundamentally different approach to chemistry thus becomes necessary, since the traditionally employed oil-based chemicals normally lack functionality. This new chemical toolbox needs to be designed to guarantee the demands of future generations at a reasonable price. The surplus of functionality in sugars and glycerol consists of alcohol groups. To yield suitable renewable chemicals these natural products need to be defunctionalized by means of dehydroxylation. Here we review the possible approaches and evaluate them from a fundamental chemical aspect.

## 1. Introduction

The concept of producing materials from renewable biomass is not new. People have been wearing woolen clothing and building wooden houses for thousands of years. Even the first artificial fibers were made from wood-derived cellulose. However, research was diverted to completely synthetic materials and chemicals due to the discovery of copious amounts of oil. Consequently, the processing of oil into chemicals has become incredibly efficient after 100 years of research, and one can reasonably argue that today's society is not only addicted to oil as a fuel, but also to its products.

However, the plentiful supply of cheap oil will diminish due to depleting reserves, while the demand for these chemicals will only grow, which will result in an increase of the price of oil. A revival of chemicals from abundantly available biomass will therefore once more become competitive with chemicals from fossil sources, and could even replace oil-derived chemicals altogether.

**Figure 1:**
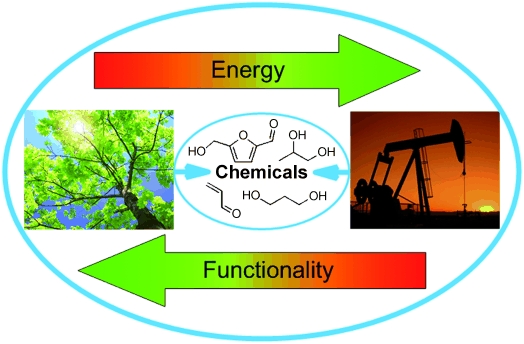
Defunctionalizing of biomass vs. functionalizing of oil.

The switch from oil-derived chemicals to bio-renewable chemicals calls for a considerable research effort because of the fundamentally different nature of the feedstock used. While the catalysts that convert oil into chemicals focus on selectively functionalizing hydrocarbons, biomass is already highly functionalized. Therefore, the catalysts that have been developed during the last 100 years are not directly applicable to biomass feedstocks. Instead, catalysts that can selectively remove some of the functionalities are desired.[1] Ideally, one should take advantage of functional groups already present in different classes of biomass. Use fatty acids for detergents, benefit from the nitrogen already present in amino acids, and treasure the hydroxy groups in sugars and polyols. In particular the conversion of carbohydrates rather than hydrocarbons is of great interest because they are so abundant, in the form of cellulose and hemicellulose (Figure 1).

This Review concerns the selective conversion of polyols. These highly oxygenated compounds can be transformed into a plethora of useful chemicals. The focus will be on the dehydration of polyols and the possible hydrogenation of the resulting double bonds. Particular attention will be paid to inducing selectivity into these processes.

## 2. Selective Dehydroxylation of Biomass

Many biomass-based materials are highly oxygenated, while most man-made chemicals are functionalized to a much lower degree. Biomass feedstock therefore needs to be deoxygenated to arrive at the same platform chemicals and final products that we currently utilize. Six approaches can be used for this essential deoxygenation:[2, 3]

1. Dehydration of vicinal diols and hydrogenation of carbonyl groups
2. Dehydration of alcohols and hydrogenation of carbon–carbon double bonds
3. Condensation of alcohols and hydrogenolysis of the resulting cyclic ethers
4. Hydrogenolysis of ethers
5. Ketonization of carboxylic acids
6. Hydrogenation of carboxylic acids

This review focuses on the selective dehydroxylation of polyols, and therefore the first three approaches will be addressed in more detail. These three methods all involve the elimination of a hydroxyl group.[4] This dehydration can proceed via elimination or through homolytic cleavage of the C–O bond on a metallic surface. The elimination reaction can proceed by either the E_1_ or the E_2_ mechanism.

**Scheme 1:**
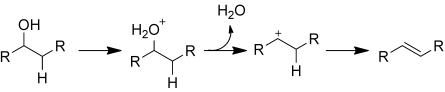
E_1_ mechanism.

The E_1_ mechanism (Scheme 1) involves protonation of a hydroxyl group, which is then expelled as water. The resulting carbocation is then neutralized by the elimination of a neighboring proton. The intermediate carbocation can be stabilized by the use of polar protic solvents, which therefore enhance reaction rates.

**Scheme 2:**
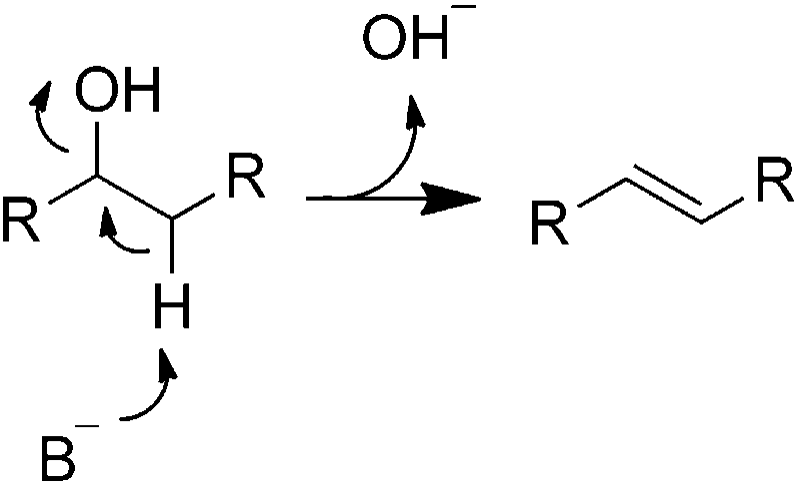
E_2_ mechanism.

Base is needed for the E_2_ mechanism (Scheme 2), whereas the E_1_ mechanism is acid-catalyzed. The presence of a carbonyl group (formed by dehydrogenation on a metallic surface) results in some acidic α-protons, which can be removed by base, resulting in E_2_ elimination.

**Scheme 3:**
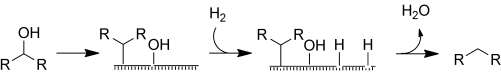
Hydrogenolysis mechanism.

Homolytic cleavage of a C–O bond can be achieved on a metallic surface (Scheme 3). Joining the two fragments with homolytically cleaved hydrogen will result in water and a dehydroxylated species. This reaction sequence is true hydrogenolysis, while this term is often used in cases where actually a sequential elimination–hydrogenation process occurs.

**Scheme 4:**
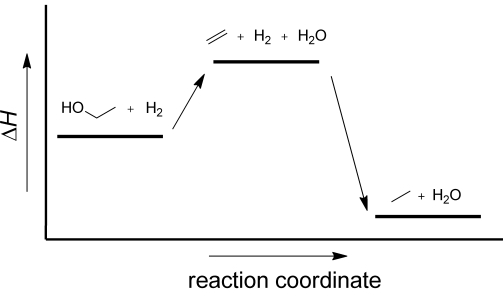
Dehydration vs. hydrogenation; endotherm vs. exotherm.

One method to introduce selectivity into the overall process is to direct the reaction in such a manner that it occurs only via one specific dehydration pathway. Because each of the three above-mentioned pathways requires different reaction conditions, this is relatively straightforward. Another point of consideration is the endothermic character of a dehydration versus the exothermic nature of an hydrogenation (Scheme 4). These opposing needs for heat can be detrimental for product selectivity. The relatively high temperature needed for initial dehydration can cause degradation of glycerol, reaction intermediates, or reaction product. The selectivity of deoxygenation can also be influenced by using additives in the catalytic system, regardless of the mechanism via which dehydroxylation occurs. These additives can coordinate available hydroxyl groups, thereby either activating or protecting the C–O bond.[5]

An example is the use of boric acid to stabilize intermediates, thereby enabling the isomerization of glucose into fructose.[6] The initial formation of a borate ester lowers the overall activation energy, thereby making intermediates readily accessible.

### 2.1 Dehydration of vicinal diols and hydrogenation of carbonyl groups

**Scheme 5:**
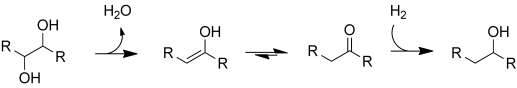
Dehydration of vicinal diols and hydrogenation of carbonyl group.

The dehydration of vicinal diols results in an enol. This readily tautomerizes into a keton or aldehyde, which is subsequently hydrogenated (Scheme 5). The keto–enol tautomerization stabilizes the system, which makes the dehydration of diols relatively easy. The challenge is to selectively eliminate either a primary or a secondary alcohol. This is for example the basis for 1,2-propanediol and 1,3-propanediol selectivity in the transformation of glycerol. It is easier to eliminate a secondary alcohol via an acid-catalyzed E_1_ mechanism, because the intermediate secondary carbocation is more stable. However, the resulting aldehyde is less stable than the ketone formed after elimination of a primary alcohol. A Brønsted acid will help in eliminating a secondary alcohol, whereas a Lewis acid can more easily coordinate to a primary alcohol, thereby weakening the C–O bond.[7] Indeed, primary alcohols are more reactive than secondary ones; this implies also a different order of reactivity if non-acidic conditions are applied.

### 2.2 Dehydration of alcohols and hydrogenation of carbon–carbon double bonds

**Scheme 6:**
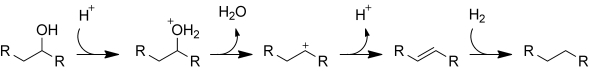
Dehydration of alcohols and hydrogenation of carbon double bond.

Isolated alcohols can be eliminated as well, which can be either acid- or base-catalyzed. However, lack of isomerization possibilities makes this process more difficult than the dehydration of vicinal diols (Scheme 6). The proximity of a double bond will offer some stabilization through a π-conjugated system, as is the case in acrolein formation.

### 2.3 Condensation of alcohols and hydrogenolysis of the resulting cyclic ethers

**Scheme 7:**
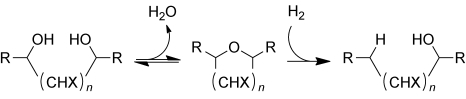
Condensation of alcohols and hydrogenolysis of cyclic ether.

This reaction sequence starts with the condensation of two alcohols by forming a cyclic ether (Scheme 7), which can then be cleaved by hydrogenolysis. However, often the rehydration and subsequent elimination (discussed in Sections 2.1 and 2.2) is faster than a subsequent hydrogenolysis. Nevertheless, the hydrogenolysis of the ether bond offers a good opportunity to introduce selectivity into the system, as studied by Koso et al.[8, 9] This group specifically studied the hydrogenolysis of tetrahydrofurfuryl alcohol and tetrahydropyran methanol using rhodium catalysts. They found that the addition of tungsten, rhenium, and molybdenum increased both the activity and the selectivity of the hydrogenolysis.[8] This higher selectivity is explained by both a smaller rhodium ensemble on a MO_*x*_-Re catalyst and coordination of the free alcohol towards the MO_*x*_.[8] This forms an alkoxide and the neighboring C–O bond is then cleaved by hydroxides present on the adjacent rhodium surface. The same bimetallic system is also highly selective in the hydrogenolysis of tetrahydropyran methanol into 1,6-hexanediol. An X-ray absorption near edge structure (XANES) and extended X-ray absorption fine structure (EXAFS) study showed that the rhodium and ReO_*x*_ particles have a direct interaction and it is indicated that the rhodium surface is covered by small ReO_*x*_ species.[9]

This reaction sequence is not applicable to glycerol, since the condensation of alcohols in order to form cyclic ethers is limited to five-membered (or larger) ring systems.

## 3. Glycerol

Glycerol is the smallest polyol readily available from biomass. It functions as the backbone of triglycerides, which constitute approximately 10 % of total biomass. Glycerol is released as a byproduct from biodiesel production. For every tonne of biodiesel produced, 100 kg of glycerol is generated. Consequently, glycerol constitutes 1 % of total biomass. Glycerol is a popular starting material for further chemical derivatization. Although this Review focuses on catalytic dehydration and reduction, a range of products can be obtained through oxidation, esterification, and etherification, stressing the versatility of this building block.[10, 11]

Jeroen ten Dam was born in the Netherlands in 1982. He obtained his M.Sc. in Organic Chemistry at the Radboud University (Nijmegen, the Netherlands). Currently, he is pursuing his PhD at the Biocatalysis and Organic Chemistry laboratory in collaboration with the Catalysis Engineering laboratory at the Technische Universiteit Delft. His main research topic is the selective production of chemicals from glycerol. He enjoys looking at catalysis from an organic synthesis point of view.

Ulf Hanefeld was born in 1966 in Germany, and grew up in then (West) Berlin and London. In 1993 he received his PhD from the Georg-August-Universität zu Göttingen, having performed the research both in Göttingen (Prof. H. Laatsch) and Seattle (Prof. H. G. Floss). After postdoctoral years with Prof. C. W. Rees (Imperial College London), Prof. J. Staunton (Cambridge) and Prof. J. J. Heijnen and Dr. A. J. J. Straathof (TU Delft), he took up a position in Delft. His research at the Technische Universiteit Delft focuses on enzymes and heterogeneous catalysis in organic synthesis.

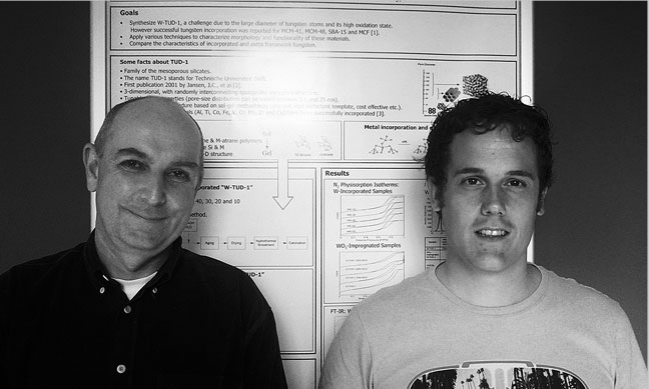

**Scheme 8:**
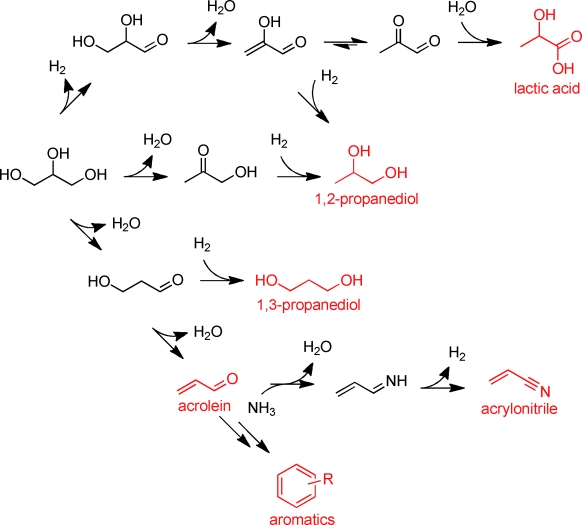
Dehydrated products from glycerol.

**Scheme 9:**
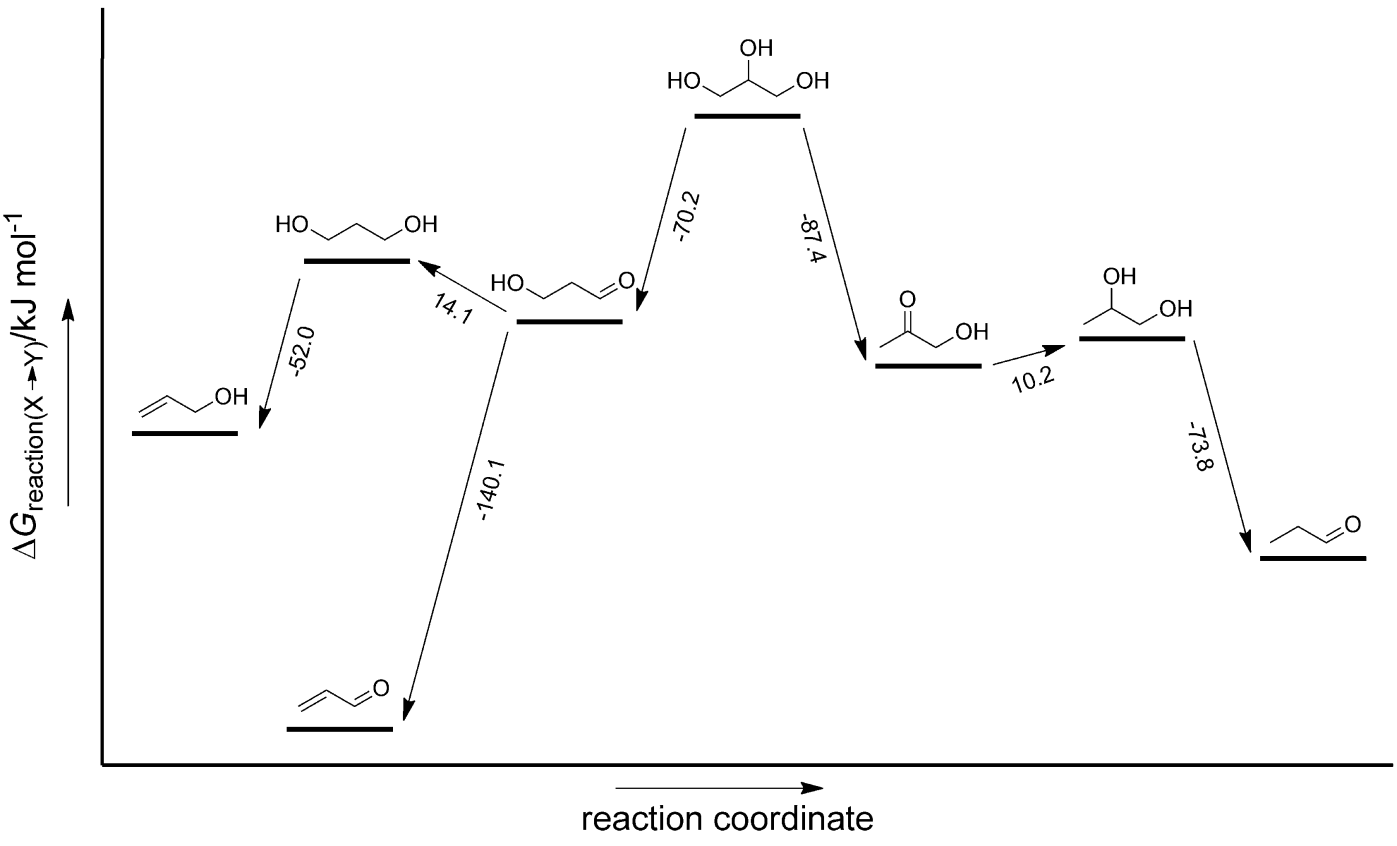
Reaction energies for glycerol to 12PD, 13PD, acrolein, their intermediates and degradation products.[4]

Scheme 8 shows the dehydration and dehydroxylation routes, leading to different products. Some of the intermediates are branching points. By choosing the appropriate catalysts and conditions, selectivities can be directed towards either of these products. The processes that lead to the molecules depicted in red will be discussed in more detail in the following chapters. Scheme 9 shows that the initial dehydration to form acetol is thermodynamically favored over the formation of 3-hydroxypropanal. Moreover, whenever 3-hydroxypropanal is formed, the subsequent dehydration to form acrolein is thermodynamically more likely than hydrogenation to 1,3-propanediol. This exemplifies the difficulties in achieving high 1,3-propanediol selectivities and it can be deduced that the formation of 1,3-propanediol is kinetically controlled.[4]

### 3.1 Glycerol to 1,2-propanediol

Most of the propylene glycol, or 1,2-propanediol (12PD), is produced by the hydration of propylene oxide. This is produced via either the chlorohydrin process or the hydroperoxide process from oil-derived propylene.[12] 12PD is primarily used as a monomer in polyesters and as an antifreeze or cooling liquid. A sustainable production starting from glycerol would involve a reduction step, rather than an oxidation, exemplifying the challenge sketched in Figure 1.

#### 3.1.1 Reaction mechanism

**Scheme 10:**
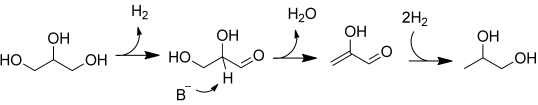
12PD formation from glycerol under alkaline conditions.

Understanding the reaction mechanism is a first step in rationally designing functional catalysts. Several reaction mechanisms have been proposed for a glycerol reduction to 12PD. Although most contributions mention hydrogenolysis, suggesting a direct C–O bond cleavage by hydrogen on a metallic surface, the actual mechanism involves an elimination followed by a reduction step, as described above (Section 2.1). Under alkaline conditions, 12PD is being formed via glyceraldehyde, through an initial dehydrogenation followed by water elimination and finally two reduction steps (Scheme 10).[13] The dehydrogenation as an oxidation seems surprising at first glance. It does however greatly ease the elimination step, as a conjugated system is obtained.

**Scheme 11:**
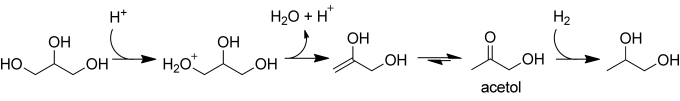
12PD formation from glycerol under acidic conditions.

Under acidic conditions, acetol is generally accepted as the key intermediate in 12PD formation (Scheme 11).[14, 15] Acetol can be formed via direct dehydration of glycerol and subsequent keto–enol tautomerization. Then it is reduced to 12PD. In principle, the acid-catalyzed elimination can eliminate either a secondary or a primary alcohol, leading to 3-hydroxypropanal or acetol, respectively. The elimination of a secondary alcohol proceeds via a relatively stable intermediate secondary carbocation and is therefore kinetically controlled. The elimination of a primary alcohol forms acetol, which is the thermodynamically more stable compared to 3-hydroxypropanal (Scheme 9). This consideration is the basis for 1,2-propanediol selectivity versus 1,3-propanediol selectivity.[16]

A complete hydrogenolysis reaction scheme is modeled for a Ru-Re/C catalyst in a batch slurry reactor. This shows that the reaction is kinetically controlled. This justifies the conclusion that hydrogenolysis can be improved by altering transition energies, by developing the appropriate catalytic system.[17]

Zhou et al. derived a kinetic model for a Cu–ZnO–Al_2_O_3_ catalyst. They showed that the reaction proceeds over two different catalytic sites. Glycerol, acetol, and 12PD are adsorbed on one catalytic site and dissociative hydrogen adsorption occurs on the other. The model showed that dehydration of adsorbed glycerol to acetol is slower than hydrogenation of acetol to 12PD and is therefore the rate limiting step. This fact will prove helpful in developing more active catalysts.[18]

#### 3.1.2 Alkaline conditions

Under alkaline conditions selectivities towards 12PD are generally high, however, the objective is to obtain high conversions as well. Vasiliadou et al. showed that the copper particle size plays a key role in silica supported copper catalyst activity.[19] Similarly, Bienholz et al. explained the essential role of copper surface area for catalyst activity for both the dehydration and hydrogenation step in the 12PD formation scheme, which makes the development of stable copper catalysts with high surface area highly desirable. Their Cu/SiO_2_ catalyst is a good example, showing excellent conversion and high 12PD selectivity (Table 1, entry 1).[20]

**Table 1:** 1,2-Propanediol from glycerol

Entry	Catalyst	Additive	Additive/metal ratio	*P* [bar]	*T* [K]	TOF[c] [h^−1^]	Conversion [%]	Selectivity to 12PD [%]	Selectivity to EG [%]	Yield of 12PD [%]	Ref.
Alkaline catalysts											
1	Cu/SiO_2_[e]	–	–	15	528	13.2	100	87.0	4.0	87.0	[20]
2	CuO/ZnO	Zn	2[a]	50	473	9.0	46.0	90.0	1.0	41.4	[21]
3	Cu/ZnO/Ga_2_O_3_	–	–	50	493	18.4	96.0	82.0	2.0	78.7	[22]
4	Cu-ZnO	Zn	1[a]	42	473	0.3	22.5	83.6	10.7	18.8	[23]
5	Cu-ZnO	Zn	1[a]	60	473	0.9	75.0	93.9	5.5	70.4	[15]
6	Pt/hydrotalcite	–	–	30	493	37.2	92.1	93.0	3.9	85.7	[24]
7	Cu/MgO	–	–	30	453	1.0	72.0	97.6	1.3	70.3	[25]
8	Cu/MgAlO	–	–	30	453	2.7	80.0	98.2	1.0	78.6	[26]
9	CuO/SiO_2_	–	–	90	473	2.6	73.4	94.3	3.6	69.2	[27]

Acidic catalysts											
10	Ru/C	Amberlyst 15	2[b]	80	393	16.8	12.9	55.4	12.9	7.1	[28]
11	Ru/C	Amberlyst 70	19[a]	80	453	804	48.8	70.2	8.3	34.3	[29]
12	Ru/C	Amberlyst 15	2[b]	80	393	38.3	21.3	76.7	–	16.3	[30]
13	Ru/C	Nb_2_O_5_	2[b]	60	453	20.0	62.8	66.5	21.2	41.8	[31]
14	Ru/TiO_2_	–	–	60	453	33.1	46.0	63.0	19.0	29.0	[32]
15	Cu/Al_2_O_3_	–	–	15	473	9.2	41.9	93.3	–	39.1	[33]
16	CuAg/Al_2_O_3_	Ag	0.4[a]	15	473	6.1	27.0	96.0	–	25.9	[34]
17	Cu-STA/Al_2_O_3_[e]	STA	1[b]	60	513	64.0	90.1	89.7	4.2	80.8	[35]
18	CuCr_2_O_4_	Cr	2[a]	80	493	6.3	80.0	84.0	–	67.2	[36]
19	Cu:Al	Al	1[a]	70	513	4.9	76.0	89.0	–	67.6	[37]
20	Ru-Cu/TMG-bentonite	Cu	0.3[a]	80	503	9.5	100	85.4	7.6	85.4	[38]
21	Ru/Al_2_O_3_	Re_2_(CO)_10_	0.5[a]	80	433	21.5	53.4	50.1	7.8	26.8	[39]
22	RuRe/SiO_2_	Re	1[b]	80	433	20.5	51.7	44.8	6.4	23.2	[40]
23	RuRe/ZrO_2_	Re	1[b]	80	433	23.7	56.9	47.2	4.0	26.9	[40]

Miscellaneous catalysts											
24	Cu/Al_2_O_3_[e]	–	–	1	473-393	0.4	100	96.7	1.9	96.7	[41, 42]
25	Pt/NaY	–	–	1[d]	503	15.0	85.4	64.0	–	54.7	[43]
26	Ru/Al_2_O_3_ and Pt/Al_2_O_3_	–	–	14[d]	493	13.7	50.1	47.2	6.3	23.6	[44]
27	Raney Ni	–	–	1[d]	453	1.0	100	25.0	32.0	25.0	[45]

[a] On a molar basis.

[b] On a weight basis.

[c] Turnover frequency (mmol_12PD_ mmol_metal_^−1^ h^−1^).

[d] N_2_ pressure and in situ hydrogen formation.

[e] Continuous reaction

The sintering of CuO in a CuO–ZnO catalyst during reaction was recently described by the same group. The sintering is caused by reaction water and this has a far greater effect than increasing reaction temperature (Table 1, entry 2).[21] The authors were able to counteract the detrimental effect of water by the co-precipitation of gallium with their CuO–ZnO catalyst. The stabilizing effect of gallium is attributed to a physical separation of the copper particles by Ga_2_O_3_ or ZnGa_2_O_4_ particles. The resulting Cu/ZnO/Ga_2_O_3_ catalyst is stable for several catalytic runs and shows high conversion and 12PD selectivity (entry 3).[22]

In a preliminary study, Liu et al. demonstrated that the performance of a co-precipitated CuO–ZnO catalyst depended on the copper particle size. Smaller particles led to higher selectivity and activity, and the sintering of these particles has to be avoided.[23] They showed that it was possible to stabilize the copper particles by a pre-reduction step, yielding a Cu–ZnO catalyst, thereby preventing the adverse effect of water. The reduction of the copper before the reaction increased the 12PD selectivity from 29 % to 84 %, while maintaining a similar conversion (Table 1, entry 4).[23]

In a follow-up paper the catalyst was described in more detail and an increase in selectivity to 94 % was reported by increasing the hydrogen pressure from 4.2 mPa to 6.0 mPa. The activity of the catalyst could be increased by using higher glycerol concentrations, temperatures, and pressures. However, these activities are reported as turnover frequencies (TOFs), recorded at ca. 25 % conversion, which makes it difficult to compare the results with other studies. 75 % conversion could be reached in 6 h, with retention of 12PD selectivity, by increasing the catalyst loading to 2.2 g (Table 1, entry 5).[15] Interestingly, the same authors reported that 12PD was formed via the glyceraldehyde mechanism, which involves the initial dehydrogenation of glycerol. The increased TOF at elevated pressures indicates that this dehydrogenation step is not rate-limiting. Therefore, a more-alkaline support (to provide subsequent dehydration) or more-active hydrogenation catalyst can improve the activity.

Hou et al. investigated platinum catalysts on various supports. Alkaline supports showed the highest activity. Platinum on hydrotalcite showed a conversion of 92 % and a 12PD selectivity of 93 %. It was superior to MgO>Al_2_O_3_>HBeta∼HZSM-5 (Table 1, entry 6).[24] Because platinum on MgO showed a reasonable conversion (50 %), this group also tried immobilization of the more economical copper on MgO. They showed that co-precipitation is more successful than impregnation. This was attributed to the better dispersion of copper particles on a co-precipitated catalyst. The catalyst showed high selectivity towards 12PD (97.6 %) and a conversion of 72 % could be reached in 20 h. The addition of NaOH promoted the dehydration step and this increased the conversion to 82 % (Table 1, entry 7).[25]

Copper supported on a hydrotalcite-like material (Cu/MgAlO) proved to be both active and selective for 12PD production (Table 1, entry 8). The high activity was ensured by a homogeneous dispersion of copper in the solid base matrix. Here, the addition of NaOH could increase the conversion of glycerol to 91 %, while only slightly decreasing the 12PD selectivity.[26]

Xia et al. prepared a CuO/SiO_2_ catalyst by the precipitation-gel technique. This catalyst showed a similar selectivity towards 12PD (94 %) as an impregnated CuO/SiO_2_ catalyst. However, the precipitation-gel catalyst was more stable due to strong copper–support interactions and much more active (73 % conversion) due to a high copper dispersity and smaller metal particle size (Table 1, entry 9). When the PG catalyst was run in a fixed-bed flow reactor, the conversion was increased to ca. 80 % and the catalyst was stable for 200 h. Even the selectivity was increased (98 %), due to limited degradation possibilities in a continuous-flow reactor.[27] This extraordinary stability is partly due to the presence of some sodium on the catalyst. This artifact of the precipitation-gel technique (which involves the addition of 4 m NaOH to a solution of Cu(NO_3_)_2_ to form a precipitate) retards the leaching of copper.[14]

#### 3.1.3 Acidic conditions

As discussed above the elimination step can be acid- or base-catalyzed. In an exploratory study Tomishige et al. showed that the sulfonic acid resin Amberlyst 15 is a more effective additive for promoting 12PD formation than homogeneous sulfuric acid and hydrochloric acid. Moreover, hydrochloric acid inhibited the catalyst activity by poisoning the ruthenium surface of the Ru/C catalyst. The activity of the Ru/C and Amberlyst 15 system was limited to 13 % conversion (giving 55 % 12PD selectivity) for a 20 wt % aqueous glycerol solution at 393 K, and could not be increased by applying higher temperatures because of the low thermal stability of Amberlyst 15 (Table 1, entry 10).[28] This limitation could be overcome by using the more thermostable resin Amberlyst 70. Temperatures up to 453 K could be applied. This, in combination with a pre-reduction step of the ruthenium particles, increased conversion to 49 % and 12PD selectivity to 70 %, with a remarkable TOF of 804 per hour (entries 11 and 12).[29, 30]

Lingaiah et al. used Ru/C in combination with several thermally stable solid acids. They showed that conversion could be linearly correlated to the number of acid sites on the solid acid. Nb_2_O_5_- and ZrO_2_-supported phosphotungstic acid, possessing moderate acid sites, proved to be the most active additives, whereas the type of additive did not influence the selectivity. The reaction conditions for Ru/C and Nb_2_O_5_ could be optimized to 63 % conversion and 67 % 12PD selectivity (Table 1, entry 13).[31] In another paper by the same authors, ruthenium was immobilized on Lewis-acidic TiO_2_ support through a deposition–precipitation method. In this way, the acid sites are in close proximity of the hydrogenating metallic particles. This approach resulted in a very stable catalyst that gave 46 % glycerol conversion and 63 % 12PD selectivity in only 8 h (entry 14).[32]

The activity and selectivity of copper immobilized on various aluminum-containing acidic supports was investigated by Zhang et al. Pure alumina was more active than the more-acidic zeolitic supports (Hβ, HY, HZSM-5, and 13X), which showed minimal or no activity at all. This could be due to the preferred selectivity of the zeolites towards acrolein, or because of the strong CuO–support interaction due to the strong acidity of the zeolites. This interaction might prevent the pre-reduction of CuO to active metallic copper. The Cu/Al_2_O_3_ catalyst was able to convert pure glycerol into 12PD at a conversion of 42 %. Interestingly, no glycerol condensation products were formed using pure glycerol and 12PD selectivity was as high as 93 % (Table 1, entry 15).[33] By co-impregnation of Cu/Al_2_O_3_ with silver, the copper could be reduced at reaction temperature, thereby rendering the pre-reduction step obsolete. Although the bimetallic catalyst showed improved 12PD selectivity, the activity of the CuAg/Al_2_O_3_ catalyst was lower compared to the parent pre-reduced Cu/Al_2_O_3_ (entry 16)_._[34]

#### 3.1.4 Catalyst promotion

A commonly used strategy to improve catalyst activity is co-immobilization of a second metal or acid with the hydrogenating metal. Sun et al. impregnated silicotungstic acid and copper on alumina. The resulting catalyst was tested in a fixed-bed reactor. The co-impregnated acid in combination with the continuous reactor resulted in good glycerol conversion (90 %) and 12PD selectivity (90 %). Moreover, the catalyst was shown to be stable with regard to activity and selectivity for 250 h (Table 1, entry 17).[35]

Yi et al. used chromium to promote the activity of copper. Chromium itself showed minimal catalytic activity,[36] and impregnation of Cr_2_O_3_ with copper did not significantly improve the catalytic properties.[46] However, by co-precipitating chromium with copper both activity and selectivity were increased tremendously. Using this method of preparation, an acidic CuCr_2_O_4_ spinel was formed. These spinels are known for storing hydrogen within their structure, thereby increasing their hydrogenation activity. This resulted, in combination with the improved acidity, in a hydrogenolysis catalyst that converted 80 % of glycerol into 12PD at 84 % selectivity (Table 1, entry 18).[36] Chromium however, is not an environmentally friendly metal. Therefore, efforts were directed towards its replacement with a more benign alternative. Aluminum could be an option. A co-precipitation Al–Cu catalyst showed higher acidity compared to two commercial copper chromate catalysts, which is translated in higher glycerol conversion. The conversion could be increased up to 76 % at 513 K, while maintaining a high 12PD selectivity of 89 %, showing only a small amount of degradation of glycerol into ethylene glycol (EG) at this temperature (entry 19).[37]

A very active and stable bimetallic catalyst was prepared by depositing ruthenium and subsequently copper on a bentonite carrier. Before the deposition of the metal, the sodium cations on the carrier were exchanged by the cations of the ionic liquid tetramethylguanidiniumlactate. These cations (tetramethylguanidinium; TMG) proved to stabilize the metal particles by strong coordination. Aggregation of the particles was prevented by strong electrostatic interactions of TMG with the negative charge in the silicate layers of the bentonite. TMG not only stabilized the metal particles, but also increased the amount of liquid product. The ruthenium in this bimetallic catalyst provides the activity (100 % conversion), while copper suppresses the degradation of glycerol into EG, thereby accommodating high 12PD selectivity (85 %; Table 1, entry 20).[38]

The influence of rhenium on the activity of several heterogeneous ruthenium catalysts was investigated by He et al. The addition of heterogeneous Re_2_(CO)_10_ was most effective in combination with Ru/Al_2_O_3_. Together with this catalyst it not only increased the conversion of glycerol, it also improved 12PD and 13PD selectivity, by preventing the degradation of glycerol into EG. In combination with Ru/C the rhenium improved both conversion and selectivity, but to a somewhat lesser extent. The mix of Ru/ZrO_2_ and Re_2_(CO)_10_ mainly improved the selectivity towards propanediols, while the effect on the conversion of glycerol was less pronounced (Table 1, entry 21). Interestingly, reasonable conversions were established at a relatively low temperature of 433 K and rhenium showed some increase in 13PD selectivity, a more valuable diol.[39] This promising lead was followed up by impregnating the rhenium and ruthenium directly on different acidic supports. Conversions between 52 and 57 % were reached using bimetallic SiO_2_, ZrO_2_, H-ZSM5, and Hβ catalysts, which were higher than when using rhenium as an additive. However, the selectivity towards 12PD was only improved for RuRe/SiO_2_ and was not improved for the other catalysts (entries 22 and 23).[40] The pretreatment of the RuRe/SiO_2_ catalyst was also investigated. High prereduction temperatures were not necessary and were even decreasing the glycerol conversion, because these high temperatures caused the metals to sinter. However, prereduction at 473 K did show higher 12PD selectivity compared to the calcined catalyst. This means that for an active and selective catalyst, the rhenium could be in a ReO_*x*_ form, while ruthenium should be in its metallic form.[47] An explanation for the promoting effect of rhenium is the surface acidity of ReO_*x*_. This would promote the dehydration step in the hydrogenolysis of glycerol. Interestingly, it also showed some enhancement in 13PD selectivity, a more valuable diol than 12PD. This effect will be discussed in more detail in the chapter concerning the formation of 13PD.

#### 3.1.5 Temperature gradient

**Scheme 12:**
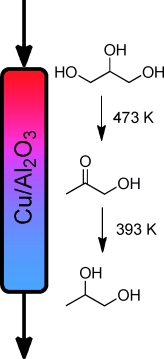
Temperature gradient reduction of glycerol.

Hydrogenolysis is not only optimized by developing the most active and selective catalysts: reaction engineering can also contribute substantially. This is already apparent from the contradicting temperature needs for dehydration and hydrogenation. Dehydration is endothermic and proceeds more rapidly at higher temperatures, while exothermic hydrogenation can be performed at milder temperatures, thereby avoiding degradation of glycerol or reaction products. Sato et al. were able to improve the hydrogenolysis of glycerol by making use of this fact. They applied Cu/Al_2_O_3_ in a fixed-bed down-flow reactor, which had different temperatures at the top and the bottom. At the top, the dehydration took place at 473 K and the subsequent hydrogenation at the bottom was performed at 393 K (Scheme 12). In this way, glycerol was completely converted and 12PD selectivity was 97 % (Table 1, entry 24).[41, 42] This is a significant increase in 12PD formation, since using Cu/Al_2_O_3_ at a constant 463 K yielded only 80 % 12PD.[41]

#### 3.1.6 Absence of H_2_

Externally added hydrogen is necessary for all previously mentioned processes to form 12PD. Normally, this hydrogen is derived from fossil fuels. However, the aqueous phase reforming of glycerol over platinum catalysts is known also. The hydrogen generated by this process can be directly used for the formation of 12PD. This was shown for the first time by D'Hondt et al. They impregnated NaY zeolite with platinum, which was able to convert 85 % glycerol with 64 % 12PD selectivity (Table 1, entry 25).[43]

Roy et al. investigated the idea of in situ generation of hydrogen in more detail. A combination of both Ru/Al_2_O_3_ and Pt/Al_2_O_3_ was more active than the individual catalysts for in situ hydrogen formation and subsequent 12PD formation. An optimal temperature of 493 K was found (conversion 50 %, 12PD selectivity 47 %; Table 1, entry 26). Increasing the temperature would further increase glycerol conversion. However, this would result in more gaseous products at the expense of 12PD selectivity. Counter-intuitively, addition of external hydrogen was detrimental to 12PD selectivity and far more methane was formed, through methanation of carbon dioxide.[44]

An interesting development was reported by Yin et al. They used Raney nickel for in situ hydrogen formation, thereby preventing the use of precious and scarce platinum and ruthenium. Raney nickel was able to catalyze the complete conversion of glycerol in 1 h at 453 K. However, it was also very active in C–C bond cleavage, resulting in substantial EG selectivity (32 %) on top of 12PD selectivity (25 %) (Table 1, entry 27).[45]

To conclude, 12PD can generally be formed with good to very good selectivities, while in many examples the conversion needs improvement. It was shown that the abundantly available copper can achieve both. By utilizing the advantages of a continuous reactor, the initial dehydration of glycerol and subsequent hydrogenation of the formed acetal can be achieved at their respective optimum temperatures. This streamlines the overall reaction and yields almost 100 % 12PD.

### 3.2 Glycerol to 1,3-propanediol

#### 3.2.1 Introduction

1,3-propanediol (13PD) is the commercially most interesting hydrogenolysis product of glycerol. It is used in resins, engine coolants, dry-set mortars, water based inks, but most of it is used in the production of polypropylene teraphthalate (PPT), which is a polyester synthesized from 13PD and teraphtalic acid. It is marketed by DuPont as SORONA. Current production methods of 13PD are catalytic routes that use oil derivatives such as ethylene oxide or acrolein as starting material. Ethylene oxide is converted to 13PD by subsequent hydroformylation and hydrogenation, whereas acrolein is subsequently hydrated and hydrogenated.[48] It is also possible to convert glycerol, or glucose, into 13PD using a fermentation process.[48, 49] It is even possible to combine 13PD and hydrogen production from crude biomass-derived glycerol, by using a mixed culture. In this way, optimal use is made of glycerol biomass, without the need for prior purification.[50]

In this review we will concentrate on the formation of 13PD from glycerol, using heterogeneous catalytic systems. The fact that the number of papers that report selective formation of 13PD is greatly outnumbered by the number of papers reporting on selective 12PD formation is a clear sign that the formation of 13PD is more challenging. However, especially over the last two years, the factors that are important to influence 13PD formation are slowly being unraveled. An overview is given in the following section. It will become clear that while for the conversion of glycerol into 12PD the activity is the main challenge, the main issue for 13PD formation is to achieve high selectivity.

#### 3.2.2 Reaction mechanism

**Scheme 13:**
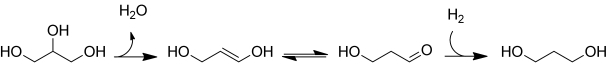
Reaction mechanism 13PD formation via dehydration hydrogenation.

The formation of 13PD proceeds via 3-hydroxypropanal as an intermediate. This aldehyde is formed after an initial elimination of the secondary alcohol group of glycerol. The formed C–C double bond undergoes a tautomerization, which yields the more stable aldehyde. Subsequent hydrogenation yields 13PD (Scheme 13). The initial alcohol elimination is endothermic and a relatively high temperature is needed for this elimination to proceed. The subsequent hydrogenation is exothermic and prefers lower reaction temperatures. Lower temperatures will also prevent further degradation of 13PD. Recently, it has been reported that 13PD is formed at lowered temperatures, using rhenium oxide as an additive. This lower temperature indicates that the dehydration hydrogenation sequence is no longer active and points in the direction of energetically more favorable direct hydrogenolysis (vide infra).

#### 3.2.3 Solvent

In 2004, Chaminand et al. were the first to report a hydrogenolysis reaction that produced more 13PD than 12PD (13PD/12PD ratio of 2). Using a slurry of Rh/C and tungstic acid in sulfolane, they were able to convert 32 % glycerol. 13PD selectivity was 12 % and a start towards 13PD was made (Table 2, entry 1).[5] This paper set the example for others to follow. The novelty of Chaminants report was both the use of tungstic acid as an additive and sulfolane as a solvent. Kurosaka et al. adopted these ideas and realized a breakthrough by impregnating several acidic supports with tungsten oxide and platinum (Table 2, entries 2–7). These catalysts were tested using 1,3-dimethyl-2-imidazolidinone (DMI) as solvent, which is considered to be more stable, polar, and aprotic than sulfolane. These acidic supports all yielded 13PD with selectivities between 26 and 39 %. TiO_2_ gave the highest selectivity (but only 17 % glycerol conversion), while ZrO_2_ stood out with a glycerol conversion of 86 %, thereby showing impressive conversion and selectivity compared to Chaminands results.[51]

**Table 2:** 1,3-Propanediol from glycerol

Entry	Catalyst	Additive	Additive/metal ratio	Solvent	Reactor type	*P* [bar]	*T* [K]	TOF[a] [h^−1^]	Conversion [%]	Selectivity to 13PD [%]	Selectivity to 12PD [%]	Yield of 13PD [%]	13PD/12PD ratio	Ref.
1	Rh/C	H_2_WO_4_	10	Sulfolane	batch	80	453	0.1	32.0	12.0	6.0	3.8	2.0	[5]
2	Pt/WO_3_/TiO_2_	WO_3_	2.9	DMI	batch	80	443	1.1	16.9	38.5	42.0	6.5	0.9	[51]
3	Pt/WO_3_/HY	WO_3_	8.6	DMI	batch	80	443	1.2	25.9	27.8	34.4	7.2	0.8	[51]
4	Pt/WO_3_/Al-MCM-41	WO_3_	8.6	DMI	batch	80	443	1.3	27.8	27.0	25.2	7.5	1.1	[51]
5	Pt/WO_3_/SiO_2_-Al_2_O_3_	WO_3_	8.6	DMI	batch	80	443	1.8	42.2	26.1	27.5	11.0	0.9	[51]
6	Pt/WO_3_/Al_2_O_3_	WO_3_	8.6	DMI	batch	80	443	2.2	43.9	30.1	25.1	13.2	1.2	[51]
7	Pt/WO_3_/ZrO_2_	WO_3_	8.6	DMI	batch	80	443	4.0	85.8	28.2	14.6	24.2	1.9	[51]
8	Pd/WO_3_/ZrO_2_	WO_3_	8.6	DMI	batch	80	443	0.8	24.0	19.6	27.5	4.7	0.7	[51]
9	Ir/WO_3_/ZrO_2_	WO_3_	8.6	DMI	batch	80	443	0.5	21.8	14.2	30.7	3.1	0.5	[51]
10	Ru/WO_3_/ZrO_2_	WO_3_	8.6	DMI	batch	80	443	0.6	46.7	7.3	19.5	3.4	0.4	[51]
11	Rh/WO_3_/ZrO_2_	WO_3_	8.6	DMI	batch	80	443	0.7	86.4	4.7	32.6	4.1	0.1	[51]
12	Pt/WO_3_/ZrO_2_	WO_3_	8.6	DMI	batch	55	443	1.9	32.5	15.2	18.2	4.9	0.8	[52]
13	Pt/WO_3_/ZrO_2_	WO_3_	8.6	Sulfolane	batch	55	443	0.8	33.8	5.3	14.6	1.8	0.4	[52]
14	Pt/WO_3_/ZrO_2_	WO_3_	8.6	EtOH	batch	55	443	2.6	38.2	23.0	13.6	8.8	1.7	[52]
15	Pt/WO_3_/ZrO_2_	WO_3_	8.6	H_2_O	batch	55	443	2.3	24.7	25.7	15.0	6.3	1.7	[52]
16	Pt/WO_3_/ZrO_2_	WO_3_	8.6	DMI-H_2_O	batch	55	443	4.2	31.6	34.9	8.7	11.0	4.0	[52]
17	Pt/WO_3_/ZrO_2_	WO_3_	8.6	DMI-EtOH	batch	55	443	4.6	45.6	29.3	18.9	13.4	1.6	[52]
18	Pt/WO_3_/ZrO_2_	WO_3_	8.6	EtOH-H_2_O	batch	55	443	3.3	45.7	21.2	8.0	9.7	2.7	[52]
19	Pt/WO_3_/ZrO_2_	WO_3_	8.6	H_2_O	continuous	40	403	4.0	70.2	45.6	2.6	32.0	17.8	[54]
20	Pt/WO_3_/TiO_2_/SiO_2_	WO_3_/TiO_2_	2.1[b]	H_2_O	batch	55	453	2.8	15.3	50.5	9.2	7.7	5.5	[55]
21	Cu-STA/SiO_2_	STA	0.033	–	continuous	5.4	483	0.2	83.4	32.1	22.2	26.8	1.4	[56]
22	Rh/SiO_2_	Amberlyst 15	–	H_2_O	batch	80	393	1.1	14.3	9.8	26.0	1.4	0.4	[57]
23	Rh-ReO_*x*_/SiO_2_	ReO_*x*_	0.5	H_2_O	batch	80	393	17.3	79.0	14.0	41.5	11.9	0.3	[58, 59]
24	Rh-MoO_*x*_/SiO_2_	MoO_*x*_	0.0625	H_2_O	batch	80	393	4.3	46.0	6.0	32.1	2.8	0.2	[58]
25	Rh-WO_*x*_/SiO_2_	WO_*x*_	0.125	H_2_O	batch	80	393	6.0	34.0	11.3	43.2	3.8	0.3	[58]
26	Ir-ReO_*x*_/SiO_2_	ReO_*x*_ and H_2_SO_4_	1	H_2_O	batch	80	393	12.0	50.0	49.0	10.0	24.5	4.9	[60]
27	Pt-Re/C	Re	1	H_2_O	batch	40	443	11.9	20.0	34.0	33.0	6.8	1.0	[61]
28	Pt-Re/C	Re	1	H_2_O	batch	40	443	5.7	45.0	29.0	27.0	13.1	1.1	[61]

[a] Turnover frequency (mmol_13PD_ mmol_metal_^−1^ h^−1^).

[b] WO_3_/Pt.

This promising lead was followed up by impregnating different hydrogenation metals on WO_3_/ZrO_2_ support. 13PD selectivities ranged from 5 to 28 % in the order Pt>Pd>Ir>Ru>Rh, while both Pt and Rh excelled with a glycerol conversion of 86 % (Table 2, entries 7–11).[51] Gong et al. used the same Pt/WO_3_/ZrO_2_ catalyst and examined the effect of the solvent (Table 2, entries 12–15). Sulfolane and DMI were used as polar aprotic solvents and EtOH and H_2_O were used as polar protic solvents. It was demonstrated that aprotic solvents were not necessary for high 13PD selectivity. On the contrary, the aprotic solvents produced more 12PD than 13PD, while the protic solvents produced 13PD at higher selectivities (23 and 26 %). The aprotic solvents showed comparable conversions (33–34 %) but were outperformed by EtOH (38 %). Conversion of glycerol in water was lagging (25 %), which can be explained by the formation of H_2_O during the reaction.[52]

EtOH/H_2_O, DMI/EtOH and DMI/H_2_O were tested as binary solvents (Table 2, entries 16 –18). Interestingly, the solvents containing DMI showed a synergistic effect for 13PD selectivity. It could be increased to 29 and 35 % using EtOH and H_2_O as the second solvent, respectively. On the other hand, the conversion increased (to 46 %) whenever EtOH was present in the binary solvent. Another interesting finding was the decrease of 12PD selectivity upon the presence of H_2_O in the binary solvent. This could increase the 13PD/12PD ratio up to 4.[52]

It seems that the initial finding of Chaminand that sulfolane, or another aprotic polar solvent, is necessary for high 13PD/12PD ratio is not general, since high ratios are also obtained in the absence of these solvents. The absence of H_2_O can increase glycerol conversion due to beneficial equilibrium conditions, but high 13PD selectivities have been shown using H_2_O or EtOH. It is likely that use of sulfolane was inspired by Bullock et al. They used sulfolane because their homogeneous catalysts were not stable in H_2_O.[53] However, this is no longer important when the switch to heterogeneous catalysts is made. Instead, results suggest that a protic solvent improves the reaction towards 13PD. This might be caused by facilitating a proton transfer from the (solid) acid to the secondary alcohol, which can then be eliminated. The polar character of the solvents is useful in stabilizing a charged intermediate. Using aqueous glycerol needs the initial elimination to work against equilibrium. However, this can be overcome by swift reduction of the formed double bond. This can be achieved by active catalysts and using favorable hydrogenation conditions. That is moderate temperatures, since hydrogenation is an exothermic reaction.

#### 3.2.4 Additives

##### Tungsten

**Figure 2:**
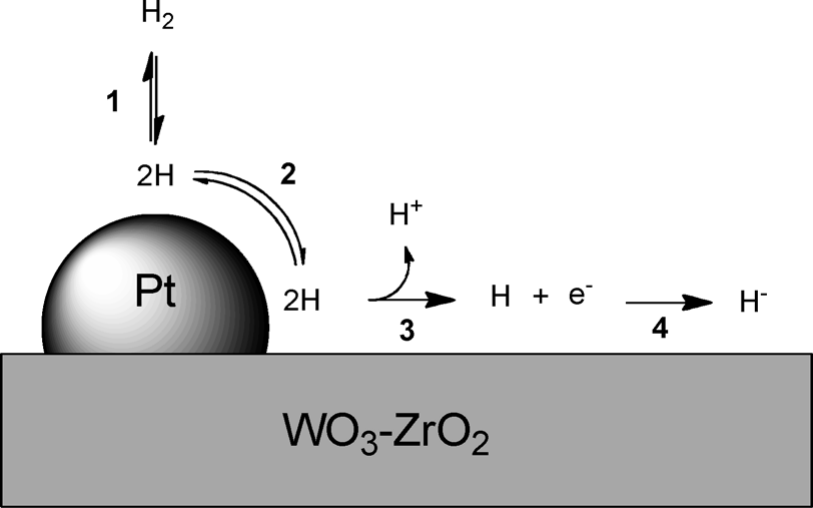
Homolytic cleavage of hydrogen on platinum and subsequent spillover.

Qin et al. also used Pt/WO_3_/ZrO_2_ as a catalyst. Using a fixed-bed reactor they were able to obtain both high conversion (70 %) and good 13PD selectivity (46 %) using aqueous glycerol at only 403 K (Table 2, entry 19).[54] The authors ascribe the low reaction temperature to the ability of the catalyst to activate hydrogen as proton and hydride. First, hydrogen is homolytically split into hydrogen atoms (**1**, Figure 2), which can spillover onto the WO_3_/ZrO_2_ surface (**2**, Figure 2). Here the hydrogen atom can donate an electron to a Lewis acidic site, generating a proton (**3**, Figure 2). This proton can be transferred to the substrate alcohol and a second hydrogen atom, acting as an acid, combines with the electron to form a hydride (**4**, Figure 2), stabilized on the Lewis acid site. This hydride is then finally used as a reductant.[62, 63] This hypothesis is supported by NH_3_ chemisorption measurements using supports that were calcined at different temperatures. Increasing the calcination temperature leads to higher acidity, resulting in higher conversion.[54]

The high 13PD selectivity can also be explained by this proton and hydride transfer mechanism over Pt/WO_3_/ZrO_2_, since protonation of secondary alcohols is preferred over primary ones. This results in an excellent 13PD/12PD ratio (Table 2, entry 19).[54] Another reason for the high 13PD selectivity could be the use of a continuous flow reactor. This will limit the degradation of 13PD formed, thereby increasing the yield.

Gong et al. found that a Pt/WO_3_/TiO_2_ catalyst showed good selectivity towards 13PD (44 %). However, activity was lagging due to the non-porous character of the TiO_2_ support. The porosity was increased by subsequently impregnating TiO_2_, WO_3_, and Pt on SiO_2_. This increased the 13PD selectivity (51 %) and doubled catalyst activity (conversion 15 %) (Table 2, entry 20).[55] It was found that TiO_2_ was responsible for a good dispersity of the platinum particles while the WO_3_ provided the Brønsted acidic sites, necessary for 13PD selectivity.

Huang et al. used the heteropoly acid silicotungstic acid (STA) as the tungsten source for their hydrogenolysis catalyst. This superacid was impregnated onto SiO_2_ to give STA/SiO_2_. In a subsequent step, copper was impregnated, to introduce the hydrogenation metal. A vapor phase reaction is necessary since STA is soluble in H_2_O and would wash out of the catalyst in case aqueous glycerol was used. This Cu-STA/SiO_2_ catalyst was tested in a vapor phase fixed bed reactor and gave high conversion (83 %) and good 13PD selectivity (32 %).[56] However, the 13PD/12PD ratio was much lower (1.4) compared to the previous catalyst, but is still very impressive for the abundantly available copper catalyst (Table 2, entry 21).

##### Rhenium

In an initial screening by Furikado et al., Rh/SiO_2_ was found to give most hydrogenolysis products from 12 catalysts tested (Rh, Ru, Pt, and Pd on SiO_2_, Al_2_O_3_, and C) at a relatively low temperature (393 K). Al_2_O_3_ was not effective under the conditions used. This was due to more demanding pre-reducing conditions of Al_2_O_3_ supported catalysts.[57] The addition of Amberlyst 15 improved the catalytic activity of Rh/SiO_2_. Earlier, the same group established this enhancing effect of the sulfonic acid resin for Ru/C.[28] However, so far both the Ru/C and Rh/SiO_2_ catalyst are more selective for 12PD, even though Rh/SiO_2_ showed 10 % 13PD selectivity (Table 2, entry 22).[57] Interestingly, a degradation study showed that 13PD is dehydrated over Ru/C, while this is limited over Rh/SiO_2_, which opens up new opportunities for a selective 13PD catalyst.[57]

To study the effect of tungsten, molybdenum, and rhenium as additives, these metals were impregnated onto Rh/SiO_2_. All three additives increase activity, but this is most pronounced for rhenium. Both rhenium and tungsten increase 13PD selectivity, while this effect is not observed for molybdenum containing catalysts (Table 2, entries 23 to 25).[58, 59]

Due to both activity and selectivity enhancement of the catalyst by ReO_*x*_, the Rh-ReO_*x*_/SiO_2_ catalyst was characterized in more detail. EXAFS analysis revealed that Re is present as Re^7+^ on the calcined catalyst, while the reductive pre-treatment reduced rhenium to an oxidation state of Re^2+^ to Re^2.5+^. This pre-treatment also resulted in a direct contact between Re and Rh and it is suggested that ReO_*x*_ clusters are in close contact to Rh particles.[58]

The authors were able to increase the 13PD selectivity tremendously by impregnating rhenium on Ir/SiO_2_. However, some additional sulfuric acid was needed, possibly because of the lower hydrogenation activity of iridium. This is unexpected, since the relatively low operating temperature suggests a direct hydrogenolysis involving the ReO_*x*_ species, while the sulfuric acid induces an elimination mechanism, which does not show 13PD selectivity.[64] Upon addition of sulfuric acid the conversion of glycerol was increased to 80 % at a 13PD selectivity of 48 %. Initial 13PD selectivity was as high as 68 % and a satisfying 13PD/12PD ratio of 5 was reached (Table 2, entry 26).[60]

Daniel et al. prepared a co-impregnated Pt–Re/C catalyst. High temperature catalyst treatment led to higher rhenium incorporation into the platinum particle with minimal metal sintering, which resulted in high 13PD selectivity (34 % selectivity at 20 % conversion) (Table 2, entry 27 and 28).[61]

**Scheme 14:**
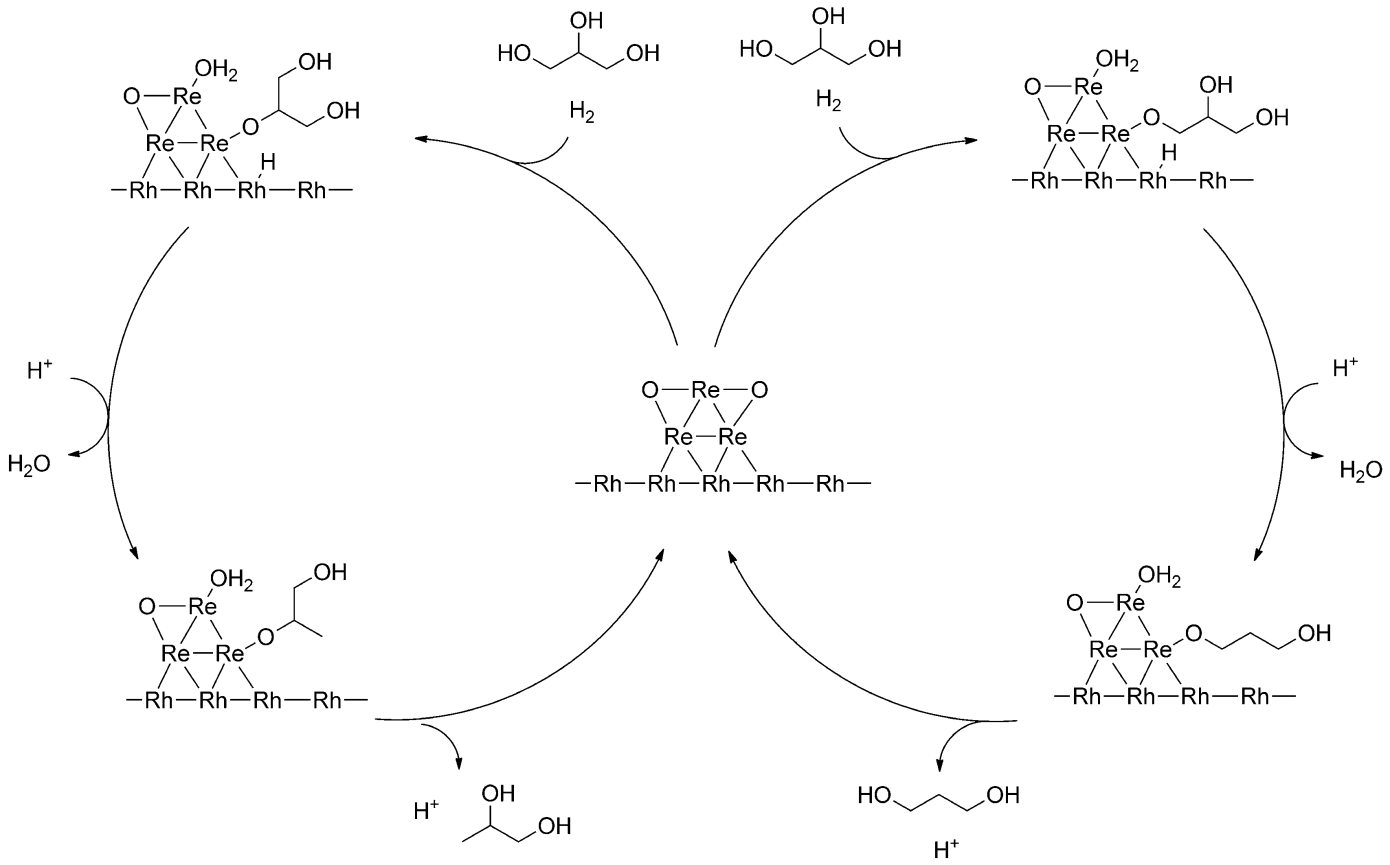
Proposed mechanism for ReO_x_ enhanced glycerol conversion and 13PD selectivity.

Tomishige et al. proposed a mechanism how rhenium oxide can promote activity and 13PD selectivity (Scheme 14).[58–60, 65] Initially, glycerol is adsorbed as an alkoxide species. Subsequently, an acidic proton transforms a hydroxyl group in a leaving group and a hydride, originating from adsorbed hydrogen, expels a water molecule. Finally, propanediol is desorbed and the initial catalyst is recovered. At this point it is not clear which factor determines either 12PD or 13PD selectivity, but it is suggested that the size of the ReO_x_ particle plays a role in steric selection.[60] It must be noted that in most examples where Re was used as an additive, 12PD selectivity is still higher than 13PD selectivity, while switching to iridium the formation of 13PD was favored over 12PD formation, reflected in the 13PD to 12PD ratio.

The formation of 13PD is not as straightforward as it seems. It is only formed under acidic conditions and in all cases 12PD formation is observed as a byproduct. In fact, in most examples where 13PD is formed, 12PD is the major product. There are a few examples where 13PD is formed as the major product and most of these processes use platinum as the hydrogenation metal and tungsten as additive. When this combination was used in a continuous reactor a 13PD yield of 32 % could be reached, while only a minimal 12PD formation was observed. Another interesting additive with respect to 13PD selectivity is ReO_x_. Using this oxide, the reaction temperature could be lowered to 393 K, suggesting a direct hydrogenolysis mechanism.

### 3.3 Glycerol to acrolein

Current industrial acrolein production is based on the oxidation of propene over BiMoO_*x*_-based catalysts.[66] Acrolein itself is mainly used as a precursor for DL-methionine synthesis. This essential amino acid cannot be synthesized by mammals and is therefore added to animal feed to accelerate growth. Acrolein production from glycerol is a promising alternative route and involves total dehydration of glycerol. Acrolein can be formed either in the liquid phase or in the gas phase. Generally, the gas phase gives higher acrolein yields. The process is normally performed in a continuous reactor, which can prevent further reactions of the reactive acrolein. An excellent recent review on acrolein from glycerol shows that high conversions and selectivities are already obtained using various catalysts, reactor types and reaction phases (Table 3, entries 1–8).[67] However, a general challenge for efficient catalysts is to decrease coke formation or circumvent catalyst deactivation in another way. By tuning the pore sizes of the catalyst, the catalyst lifetime can be extended.[68]

**Table 3:** Acrolein from glycerol

Entry	Catalyst	Additive	Time on stream [h]	WHSV[a] [h^−1^]	*P* [bar]	*T* [K]	Reactor type	Phase	Conversion [%]	Acrolein sel- ectivity [%]	Acrolein yield [%]	Ref.
1	STA/SiO_2_	–	5	n.a.	1	548	continuous	gas	100	87.0	87.0	[67]
2	Nd_4_(P_2_O_7_)_3_	–	8	227[b]	1	593	continuous	gas	87.2	79.9	69.7	[67]
3	Gd_4_(P_2_O_7_)_3_	–	8	227[b]	1	593	continuous	gas	88.2	78.9	69.6	[67]
4	Sm_4_(P_2_O_7_)_3_	–	8	227[b]	1	593	continuous	gas	89.7	77.8	69.8	[67]
5	ZSM-5	–	n.a.	n.a.	1	588	continuous	gas	98.3	74.9	73.6	[67]
6	H_2_SO_4_	–	n.a.	n.a.	345	673	continuous	supercritical	92.0	80.4	74.0	[67]
7	H_2_SO_4_	–	n.a.	n.a.	345	623	batch	liquid	55.0	86.0	47.3	[67]
8	ZnSO_4_	–	n.a	n.a.	250	633	continuous	liquid	50.0	75.0	37.5	[67]
9	WO_3_/ZrO_2_	–	10	80[b]	1	588	continuous	gas	100	65.0	65.0	[69]
10	Nb_2_O_5_	–	10	80[b]	1	588	continuous	gas	88.0	51.0	44.9	[70]
11	Nb_2_O_5_-SiO_2_	–	2	80	1	593	continuous	gas	100	65.0	65.0	[71]
12	ZSM-5	–	2	7.2	1	588	continuous	gas	93.7	57.4	53.8	[72]
13	TiAl	–	10	400[b]	1	588	continuous	gas	67.0	52.0	34.8	[73]
14	TiZr	–	10	400[b]	1	588	continuous	gas	60.0	45.0	27.0	[73]
15	STA/SiO_2_	–	5	0.6	1	548	continuous	gas	98.3	86.2	84.7	[74]
16	PTA/ZrO_2_	–	10	400[b]	1	588	continuous	gas	76.0	71.0	54.0	[75, 76]
17	CsPTA	–	1	2.8	1	548	continuous	gas	100	98.0	98.0	[7]
18	CsPTA	–	5	2.8	1	548	continuous	gas	41.0	94.0	38.5	[7]
19	Pd/CsPTA	H_2_	5	2.8	1	548	continuous	gas	79.0	96.0	75.8	[7]
20	ZrNbO	–	48	0.5	1	573	continuous	gas	99.0	72.0	71.3	[77]
21	VOHPO_4_	O_2_	10	0.5	1	573	continuous	gas	100	66.0	66.0	[78]
22	VPO	O_2_	10	0.5	1	573	continuous	gas	100	64.0	64.0	[79]
23	ZSM-5	–	n.a.	335	1	623	continuous	gas	100	62.1	62.1	[80]

[a] Weight hourly space volume (mass_glycerol_ mass_catalyst_^−1^ h^−1^).

[b] Gas hourly space volume (*V*_feed_ *V*_catalyst_^−1^ h^−1^).

#### 3.3.1 Reaction mechanism

**Scheme 15:**
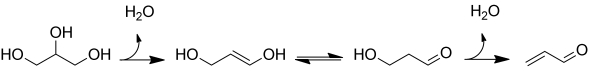
Acrolein formation mechanism.

The acrolein formation pathway closely resembles the dehydration-hydrogenation pathway of 13PD formation. Instead of hydrogenating the 3-hydroxypropanal, another hydroxyl group is eliminated, resulting in the stable π-system of acrolein (Scheme 15). Therefore it is not surprising that acidity is the key in catalyst activity and selectivity. Strong acidity will lead to higher activity. However, acrolein selectivity can decrease due to coke formation.

Chai et al. published a guiding article in which they tested numerous materials, ranging from alkaline to highly acidic catalysts. They concluded that catalysts having a Hammett acidity function (*H_0_*) between −8.2≤*H_0_*≤−3.0 were most effective in selective glycerol dehydration, giving acrolein selectivities between 60 and 70 %. Catalysts with stronger acid sites (*H_0_*≤−8.2) or weaker acid sites (−3.0≤*H_0_*≤6.8) gave lower acrolein selectivities due to coke deposition during the reaction. Their data also implied that Brønsted acidic catalysts were more effective than Lewis acidic catalysts. Alkaline catalysts were found to be ineffective for acrolein formation. Interestingly, their most successful catalyst (WO_3_/ZrO_2_) is the support material of the most successful 13PD catalyst (Table 3, entry 9).[69] This is not surprising, since the first step is identical to the 13PD formation (Scheme 13).

#### 3.3.2 Acidity and pore size manipulation to improve catalyst performance

Niobium oxide is a suitable material to show the effect of the acidity of the material on acrolein formation. The acidity of the material can be controlled by choosing an appropriate calcination temperature. A relatively low calcination temperature gives the highest amount of relevant acidic sites (−8.2≤*H*_0_≤−3.0). And indeed, these catalysts gave the highest acrolein selectivity (51 % selectivity at 88 % glycerol conversion) (Table 3, entry 10).[70] Another group was able to improve the niobium based catalyst, through impregnation on SiO_2_. Initially, the catalyst reached 65 % selectivity, at 100 % conversion, but this dropped to 50 % conversion after 10 h of operation (Table 3, entry 11).[71]

The amount and strength of acidic sites can also be tuned by changing the Si/Al ratio in H-ZSM-5. Na-ZSM-5, effectively blocking the Brønsted acidic sites, gave less than 1 % acrolein, proving the importance of this acid type.

Of the H-ZSM-5 with Si/Al ratios 30, 60, 150, 500 and 1000, the ZSM-5 150 was most effective. This implies again an optimum in acidity for acrolein formation. The best catalyst reached 94 % conversion and 57 % acrolein selectivity during the initial two hours of reaction, and after 12 h a selectivity of 46 % at 39 % glycerol conversion remained. This strong deactivation is caused by pore blocking due to coke formation (Table 3, entry 12).[72]

Obviously, tuning of acidity is not the only relevant parameter. This was demonstrated by the synthesis of bimetallic catalysts, consisting of combinations of Sn, Ti, Zr, Al, Si and Zn oxides, with different acidities. Some of the catalysts with the appropriate acidity between −8.2≤*H_0_*≤−3.0 did not perform as well as expected. This could be explained by the micro pores present in these catalysts. These small pores were easily blocked by coke formation and could retain the formed acrolein to facilitate secondary reactions (Table 3, entries 13 and 14).[73]

This effect was also noticed by Tsukuda et al. A series of SiO_2_ supported silicotungstic acids (STA) showed improved performance, both in activity and selectivity, when the pore sizes where increased from 3 to 10 nm. Their STA/SiO_2_ with an average pore size of 10 nm showed an excellent conversion (98 %) and very high acrolein selectivity (86 %) during the first 5 h of reaction (Table 3, entry 15).[74]

In another paper by Chai et al., the performance of phosphotungstic acid (PTA) on SiO_2_ and ZrO_2_ was compared. PTA on ZrO_2_ showed superior activity and selectivity and was able to produce a 54 % yield, even after 10 h on stream. This is explained by the higher stability and dispersion of the PTA on the ZrO_2_ surface, compared to the SiO_2_ carrier (Table 3, entry 16).[75, 76]

PTA can be used as a heterogeneous catalyst by preparing its insoluble cesium salt. Its initial performance is outstanding: 98 % yield during the first hour of reaction (Table 3, entry 17). However, the catalyst is prone to severe deactivation due to coking. Selectivity, on the other hand, could be maintained (Table 3, entry 18). Coking could be delayed by impregnation of the CsPTA with a small amount of palladium and co-feeding with hydrogen. In this way a conversion of 79 % accompanied by a selectivity of 96 % could be reached (Table 3, entry 19).[7]

#### 3.3.3 Catalyst deactivation

All catalysts mentioned before suffer from deactivation due to coke formation. This is unavoidable due to the acidic nature of the catalysts and the elevated temperature. Interestingly, the deactivation of a ZrNbO catalyst was very limited compared to impregnated NbO_x_/ZrO_2_ catalyst. A high glycerol conversion and acrolein selectivity could be maintained for over 48 h (Table 3, entry 20), which was explained by the formation of a solid solution of Nb into the ZrO_2_ due to the ZrNbO synthesis method.[77]

Loading CsPTA with a platinum group metal and co-feeding hydrogen, as demonstrated in a previous example, is one possibility to readily remove the formed coke from the catalyst. However, adding precious metal and the introduction of a reductive atmosphere is not ideal.

Another possibility is to oxidize the formed coke, by adding oxygen. This was done by Wang et al. over a vanadium phosphate oxide (VPO) and a vanadium oxide hydrophosphate (VOHPO_4_) catalyst. In this case, the oxygen present in the feed can also re-oxidize vanadium to its active form. By adding oxygen, the conversion was increased from 43 % to 100 % and selectivity from 35 % to 66 % (Table 3, entry 21 and 22). In theory, acrolein could also be oxidized to acrylic acid, an important monomer in polymer chemistry. However, in this case only small amounts of acrylic acid were formed.[78, 79]

A technological solution for the coking problem would be an FCC type reactor. In these reactors both catalyst and substrate are continuously fed to the reactor and separated afterwards. This allows for catalyst regeneration outside catalysis conditions. This opens up the opportunity to burn off the coke and then feed the regenerated catalyst to the reactor. This type of operation was simulated by Corma et al. using a moving bed reactor. In this reactor catalyst and substrate are fed simultaneously, however the catalyst regeneration unit and re-feeding unit is absent. This resulted in 62 % selectivity at 100 % conversion, using H-ZSM-5 as a catalyst (Table 3, entry 23). The advantage of this type of operation is that while the catalyst is regenerated by burning off the coke, the produced heat is used to maintain the process temperate.[80]

Acidity and porosity are the main parameters that influence activity and selectivity of the catalyst. These can be effectively controlled and high acrolein yields can be obtained. However, the catalysts have only a limited lifetime due to coke formation during the reaction. Interestingly, highly selective acrolein catalysts often consist of a tungsten containing heteropolyacid. These materials show 13PD selectivity in combination with a hydrogenation catalyst. This can be explained by the identical initial steps in both acrolein as well as 13PD formation.

### 3.4 Glycerol to other products

#### 3.4.1 Lactic acid

Most papers on deoxygenation of glycerol concern the formation of either 1,2-propanediol, 1,3-propanediol or acrolein. However, there are some other interesting possibilities. For instance, in case 1,2-propanediol is produced under alkaline conditions, lactic acid is often found as a side product.[81, 82]

Lactic acid is a widely used chemical in the food, pharmaceutical and chemical industries. The demand for lactic acid increases, mainly due to the use of polylactic acid as a biodegradable polymer. Currently, lactic acid is produced through the fermentation of carbohydrates. However, this process can be more efficient and other, alternative routes should be investigated.

**Scheme 16:**
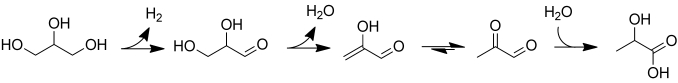
Lactic acid formation from glycerol via base catalyzed intramolecular Cannizzaro reaction.

The fact that lactic acid, having an oxidized carbon, is formed under reductive conditions seems somewhat strange at first, but the observation that this only occurs under alkaline conditions is a clue to its formation process. This resembles the 1,2-propanediol formation (Scheme 10), but instead of being reduced after dehydration, the intermediate is subjected to a disproportionation via an intramolecular Cannizzaro reaction.[83] The metallic catalyst is necessary for the initial dehydrogenation, which allows lower reaction temperatures compared to the hydrothermal formation of lactic acid (Scheme 16).[84]

**Scheme 17:**
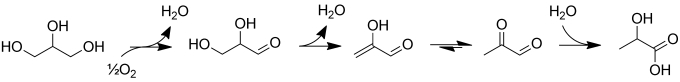
Lactic acid formation under oxidative conditions.

However, lactic acid is normally formed under oxidative conditions, which do not need high temperatures for dehydrogenation.[85] Under oxidative conditions, glycerol is readily oxidized to glyceraldehyde. The art lies in facilitating the subsequent dehydration, which will yield lactic acid, instead of oxidation, to yield glyceric acid (Scheme 17).[85]

#### 3.4.2 Aromatics

In case aromatics are mentioned in combination with biomass, one immediately thinks of lignin. Lignin is abundant in wood and its amount is only surpassed by cellulose. However, lignin consists of a mix of methoxylated monolignols and needs to be degraded into a multitude of chemicals, before it can be processed further. Such a complex feed will hinder the selective formation of useful products.

Therefore, it is an interesting development that propanal can be converted into aromatics over zeolitic catalysts.[86–88] Aromatics are supposedly formed via a series of subsequent aldol and elimination reactions. The resulting unsaturated aldehyde is then ring-closed and elimination of the final hydroxyl yields the aromatic ring system.[87] The catalyst stability can be influenced by adjusting the pore size of the H-ZSM-5 by desilication. Mildly desilicated catalyst performed best in terms of inhibiting coke formation and showed satisfying selectivity towards aromatic compounds.

Aromatics can also be formed directly from glycerol, by initial dehydroxylation or dehydration followed by the aldol condensation sequence mentioned above. Three dimensional HY and H-ZSM-5 showed aromatics formation, indicating that the pore intersections are important in aromatics formation. The aromatics yield was improved by using a dual bed Pd/ZnO–H–ZSM-5 catalytic system, by partially deoxygenating glycerol over Pd/ZnO before it is fed to H–ZSM-5.[89]

This method leads to a mixture of aromatics, which need to be purified when used as chemicals but can directly be applied as fuels, representing an interesting lead towards aromatics from biomass, without using lignin.

#### 3.4.3 Acrylonitrile

Obviously, conversion of glycerol and carbohydrates leads to compounds consisting of hydrogen, carbon and oxygen. Bio-based molecules that have nitrogen containing functional groups normally originate from amino acids. The formation of acrylonitrile from glycerol is not only an interesting reaction because a nitrogen functionality is introduced, but also because acrylonitrile is a monomer for polyacrylonitrile and starting material for acrylamide and acrylic acid.

**Scheme 18:**
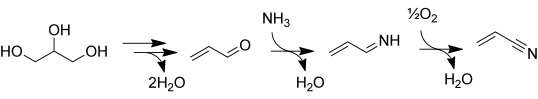
Ammoxidation of glycerol.

Glycerol can be converted (83 % conversion) into acrylonitrile (58 % selectivity) in vapor phase over a VSbNb/Al catalyst while co-fed with ammonia and oxygen. The reaction occurs in two stages. First, acrolein is formed, after which ammonia is inserted in a condensation and oxidation (Scheme 18). The vanadium provides activity in the reaction, while the antimony and niobium are important for acrylonitrile selectivity.[90]

Glycerol ammoxidation can be performed in liquid phase when promoted by microwave irradiation. In this case ammoniumhydroxide is used as the nitrogen source and hydrogen peroxide is used as a soluble oxidizing agent. An acrylonitrile selectivity of 84 % can be reached at 47 % glycerol conversion.[91]

#### 3.4.4 Epichlorohydrin

**Scheme 19:**
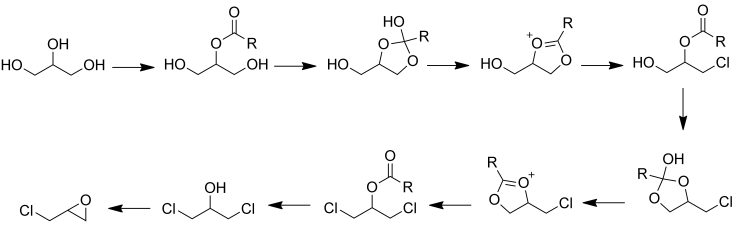
α,γ-dichlorohydrin formation from glycerol.

Before glycerol became abundantly available through the transesterification of fats, it was chemically produced via epichlorohydrin. Illustrative is the fact that nowadays epichlorohydrin is available from glycerol and is used as a component in epoxy resins. Glycerol can be selectively converted into α,γ-dichlorohydrin, using gaseous hydrochloric acid and a carboxylic acid catalyst (Scheme 19). Epichlorohydrin can be obtained by treating the dichlorohydrin with base.[92]

The selectivity towards α,γ-dichlorohydrin is a result of the carboxylic acid catalyst. This is an advantage over the less selective formation from allylchloride, since the α,γ-dichlorohydrin is 10 times more reactive in epichlorohydrin formation than its α,β-counterpart.[93, 94]

#### 3.4.5 Ethylene glycol

Ethylene glycol is often observed as a byproduct in propanediol formation from glycerol. It is used in high quantities as a starting material for PET, a polyester used in the manufacturing of drinking bottles. However, the formation of ethylene glycol is a result of a C–C bond cleavage, which is an inefficient use of glycerol feedstock and its formation has to be suppressed to allow for higher propanediol selectivities.

**Scheme 20:**
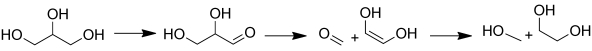
Ethylene glycol formation via retro aldol.

The formation of ethylene glycol proceeds via a retro-aldol condensation, followed by a hydrogenation (Scheme 20).[13] The formation of ethylene glycol is influenced by the type of metal that is used. For instance, higher amounts of ethylene glycol are formed upon addition of platinum to a Ni/Al_2_O_3_ catalyst.[95] Ethylene glycol formation is also more pronounced on ruthenium catalysts, while copper catalysts are normally very selective towards 12PD. These are indications that ethylene glycol can also be formed by a deformylation pathway.

## 4. Longer Chain Polyols

A considerable research effort has been directed to the conversion of glycerol. It is the smallest polyol readily available from biomass. Despite its limited size it already offers a plethora of possible products. Compared to cellulose, constituting 35–50 % of total biomass, glycerol is only a minor component. Before cellulose can be used as a starting material for chemicals, it has to be broken down into its glucose monomers. Current research focuses on heterogeneous acidic catalysts for the hydrolysis of cellulose.[96–98] One of the encountered problems is the crystallinity of cellulose. This order in the cellulose fiber hinders the accessibility of the 1,4-glucosidic bond for hydrolysis.

However, its abundance in biomass is not proportional to the amount of research focusing on the conversion of cellulose-derived glucose to chemicals. This is a result of the complexity of glucose compared to glycerol. Besides its additional hydroxyl groups, it also has an aldehyde functionality, which significantly increases the challenge of selective glucose conversion. This challenge is often met by hydrogenating glucose to sorbitol, thereby greatly reducing the reaction possibilities.

Employing an acidic catalyst, sorbitol can be converted into isosorbide via a stepwise dehydration via 1,4-anhydrosorbitol.[99] However, the protons needed for acid catalyzed dehydration can also be directly provided by high temperature water, thereby avoiding the addition of a separate acidic catalyst.[100] By combining a reduction catalyst with an acidic catalyst, cellulose can also directly be converted into isosorbide, merging the cellulose hydrolysis, glycose reduction and sorbitol dehydration into one process.[101]

A strategy employed by the group of Dumesic is to reduce the functionality of sorbitol and glucose through hydrodeoxygenation over a PtRe/C catalyst. PtRe/C is both a deoxygenating and reforming catalyst, providing the hydrogen for hydrogenation reactions. This combination of reactivity yields alkanes and a scale of mono-functional chemicals, like mono-alcohols, heterocycles, ketones and acids. Due to their extensive deoxygenation (80 % of initial oxygen removed) they phase separate from water, which facilitates the subsequent upgrading to fuel and chemicals.[102, 103]

For instance, a duel-bed catalytic system was used to convert the mono-functional chemical stream from the PtRe/C catalyst into C_7_ to C_12+_ molecules. A CeZrO_*x*_ catalyst was used for ketonization of the carboxylic acid feed, while Pd/ZrO_2_ was used as an aldol condensation and hydrogenation catalyst. The resulting C–C coupled products were finally dehydrated and hydrogenated over Pt/SiO_2_-Al_2_O_3_ to give diesel-like alkanes.[104]

C–O bond cleavage and hydrogenation are the reactions that form the basis of aqueous phase hydrodeoxygenation. Often, sometimes unwanted, C–C bond scission is also observed. Li and Huber sought to clarify the reaction pathways in aqueous phase hydrodeoxygenation over a Pt/SiO_2_–Al_2_O_3_ catalyst by studying these three reaction types. They found that C–O bond cleavage mainly occurs via dehydration over Brønsted acidic sites while C–C bond scissions involve a retro-aldol condensation or decarbonylation over Pt metallic surface. By using these three reactions they were able to construct a degradation pathway for sorbitol and they foresee that the product selectivity can be altered by tuning the activity of individual reactions.[105]

**Scheme 21:**
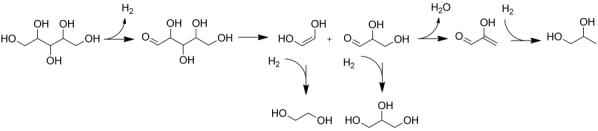
Xylitol hydrogenolysis under alkaline conditions.

In case sorbitol is treated under alkaline hydrogenating conditions it is subjected to C–C bond scission through a retro-aldol reaction and subsequent hydrogenation (Scheme 21) similar to the formation of ethylene glycol.[13] In 1958 Clark reported the formation of 12PD, ethylene glycol and glycerol using a Ni/kieselguhr catalyst and Ca(OH)_2_ as an additive.[106] Back then, the formation of glycerol was the main objective, while nowadays the formation of both 12PD and ethylene glycol is preferred.

Sorbitol can be converted into mainly 12PD using Ni/NaY as a catalyst (66 % conversion, 62 % 12PD selectivity), while Pt/NaY yields primarily glycerol (43 % conversion, 62 % glycerol selectivity). The addition of Ca(OH)_2_ increases the conversion of sorbitol in both cases (to 75 % and 60 % conversion, respectively). It is postulated that the difference in product selectivity is a result from other modes of adsorption of sorbitol over the two metals.[107]

Employing a Ru/CNF catalyst (CNF=carbon nanofiber) with Ca(OH)_2_ as a promoter, Zhou et al. were able to form ethylene glycol, 12PD, and glycerol. The benefits of using CNF instead of normal carbonaceous support are the higher dispersity of metal on the carbon surface and the limited amount of micropores in CNF, which cause mass transfer limitations.[108, 109] Similar conditions can be applied using xylitol as a starting material. This will also yield mainly 12PD, ethylene glycol and glycerol. However, lactic acid is observed as a major product as well. Sun et al. found that the activity increased using the following metals: Rh>Pd>Ru>Pt. The support of ruthenium catalysts influenced activity as well (TiO_2_>ZrO_2_>Mg_2_Al_2_O_*x*_>Al_2_O_3_>C) and lactic acid selectivity increased upon increased basicity of the support.[110]

Another interesting process is the formation of 5-hydroxymethylfurfural (HMF) by dehydration of fructose.[111] Riisager demonstrated the conversion of aqueous solutions of fructose and hydrochloric acid by microwave irradiation. The advantage of microwave irradiation lies in swift and precise heating and could thereby increase the formation rate of HMF, compared to thermal heating. The product distribution was comparable for the two heating methods, which proved microwave irradiation to be an efficient alternative heating method.[112]

In another paper by the same group, fructose was converted into HMF using boric acid as an additive. Boric acid is not sufficiently acidic by itself for fructose dehydration. However, by forming borate esters with fructose, the reaction mixture is acidified through a shift in equilibrium and HMF is readily formed. The addition of sodium chloride further improved HMF yield. This was ascribed to a salting-out effect in the two-phase system. By increasing the extraction of the formed HMF it is no longer prone to rehydration due to a shift of the equilibrium.[113]

Obviously, formation of HMF from glucose instead of fructose would be preferred from an availability point of view, since glucose can be generated from cellulose. However, this calls for the isomerization of glucose into fructose, prior to dehydration. This isomerization is catalyzed by alkaline sites, while the formed fructose is dehydrated to HMF on acidic sites. Yan et al. synthesized a SO_4_^2−^/ZrO_2_–Al_2_O_3_ catalyst that bears both alkaline and acidic sites, and were thereby able to directly convert glucose into HMF in a 48 % yield, using DMSO as a solvent.[114]

**Scheme 22:**
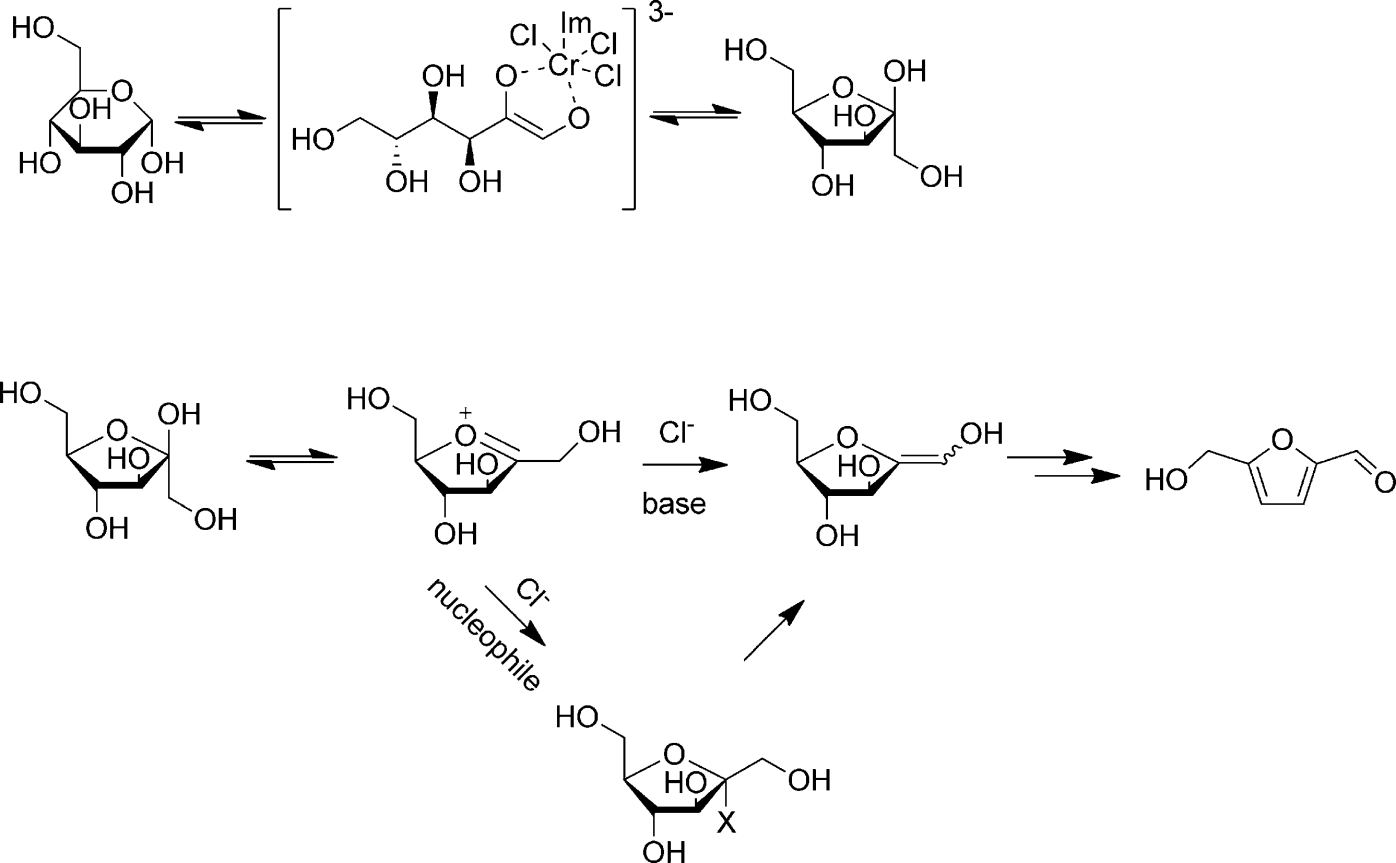
HMF formation from glucose.

Zhao et al. were able to generate a remarkable HMF yield of 68 % from glucose, by using chromium dichloride as a catalyst and an imidazolium-based ionic liquid as a solvent. The benefit of using an ionic liquid as a solvent is twofold. Firstly, HMF degradation is minimized by minimizing water interactions. Secondly, and more importantly, the imidazolium ionic liquid takes part in the reaction mechanism (Scheme 22).[115]

Despite extensive catalyst testing, only chromium showed high HMF yields. This metal is thought to form a trichloride anion, with the ionic liquid cation as a counterion. This anion facilitates the conversion of glucose into fructose which is then dehydrated to HMF.[115, 116]

Yong et al. showed that the addition of sterically hindered carbene ligands, similar to the imidazolium anion of the ionic liquid, could improve the reaction yield even more. These ligands prevent over-coordination, thereby leaving the chromium center in its most active state, yielding 81 % HMF from glucose.[117]

In a communication Binder et al. described the formation of HMF from glucose using dimethyl acetamide together with a halide salt as a solvent, thereby circumventing the use of an ionic liquid. They found that the halide played an important role in the reaction mechanism as a nucleophile, by quenching an intermediate oxonium ion in HMF formation (Scheme 22). Bromide proved to be most efficient and chromium tribromide gave a HMF yield of 80 %.^118^

Interestingly, they were also able to convert cellulose directly into HMF. Here, they used additional ionic liquid, to dissolve cellulose. Hydrochloride was added for the hydrolysis of the glucoside bonds. Overall, an impressive yield of 54 % HMF from cellulose was obtained.[118]

Another group reported the direct conversion of cellulose into HMF through the addition of RuCl_3_. The RuCl_3_ is able to efficiently hydrolyse cellulose to glucose, while CrCl_2_ is essential for a smooth conversion of glucose into fructose, which is then dehydrated to form HMF.[119]

By substituting toxic chromium dichloride with non-toxic boric acid, Ståhlberg et al. were able to convert glucose into HMF. Boric acid lowered the transition state for glucose isomerization through formation of a borate ester. The resulting fructose is subsequently converted into HMF. An HMF yield of 42 % could be obtained by using one equivalent of boric acid. Using more boric acid results in the formation of more esters, which are too stable, thereby hindering the HMF formation.[6]

## 5. Conclusion

The majority of research in deoxygenation of polyols has been invested in the conversion of glycerol. This smallest naturally available polyol can be converted into a range of useful chemicals through dehydration, direct hydrogenolysis or a dehydration-hydrogenation sequence.

The major deoxygenation products from glycerol are 12PD, 13PD and acrolein. The formation of these products all have their individual challenges. Acrolein can be formed at both high conversion and selectivity, but the catalysts used for this process suffer from deactivation through coke formation. 12PD can be formed at high selectivities using abundantly available copper as a hydrogenating catalyst and the aim is to increase the conversion. For 13PD it is still the selective formation itself that is a main concern. In this respect the catalysts used for acrolein formation might be a good lead for new catalyst developments, since both acrolein and 13PD share the same initial intermediates.

In principle, these processes can also be applied to longer chain polyols like erythritol and xylitol. However, the wide research field that exists for glycerol is missing. This could be due to the larger challenge to obtain products in a selective way, since the complexity of these C4 and C5 polyols is increased by the extension of their carbon backbone. Another reason could be the lower availability of these polyols from biomass. Erythritol is produced from glucose by fermentation and is as such not present in large quantities from biomass. However, xylose is a main constitute of hemicellulose and can be readily converted into xylitol through hydrogenation. But unlike cellulose, hemicellulose is composed of a mix of sugars, which makes purification of these sugars before derivatization desirable.

Sorbitol, on the other hand is readily available from the hydrolysis and subsequent hydrogenation of the formed glucose. The main products from glucose and sorbitol dehydration are hydroxymethylfurfural and isosorbide, respectively. Hydrogenolysis of sorbitol yields ethylene glycol, 12PD and glycerol. One might expect that a broader range of chemicals would be available from these starting materials, obviously the potential of these feedstocks has not yet been realized.
